# Isolated inflammatory involvement of the occipital artery in giant cell arteritis and polymyalgia rheumatica: findings from a retrospective analysis and the critical role of MRI in diagnosis

**DOI:** 10.1007/s00296-024-05765-4

**Published:** 2025-01-13

**Authors:** Konstanze V. Guggenberger, Lukas Riedling, Daria Kern, Rudolf A. Werner, Marius L. Vogt, Matthias Fröhlich, Marc Schmalzing, Mirko Pham, Thorsten A. Bley

**Affiliations:** 1 Department of Diagnostic and Interventional Neuroradiology, University Hospital Wuerzburg, Josef-Schneider-Strasse 11, 97080 Wuerzburg, Germany; 2 Department of Diagnostic and Interventional Radiology, University Hospital Wuerzburg, Oberduerrbacher Strasse 6, 97080 Wuerzburg, Germany; 3 Department of Diagnostic and Interventional Radiology and Nuclear Medicine, Division of Nuclear Medicine, University Hospital Goethe University Frankfurt, Theodor-Stern-Kai 7, 60596 Frankfurt am Main, Germany; 4 Department of Internal Medicine II, Rheumatology and Clinical Immunology, University Hospital Wuerzburg, Oberduerrbacher Strasse 6, 97080 Wuerzburg, Germany

**Keywords:** Vasculitis, Giant cell arteritis, Polymyalgia Rheumatica, Temporal arteritis, Magnetic resonance imaging

## Abstract

**Background:**

Diagnosis of Giant Cell Arteritis (GCA) and Polymyalgia rheumatica (PMR) may be challenging as many patients present with non-specific symptoms. Superficial cranial arteries are predilection sites of inflammatory affection. Ultrasound is typically the diagnostic tool of first choice supplementary to clinical and laboratory examination. Inflammation of temporal arteries can be detected sonographically with high reliability. However, due to the vessel’s course and location, occipital arteries evade sonographic detectability.

**Objective:**

The aim of our study was to evaluate the infestation pattern of superficial cranial arteries in GCA and PMR patients with special focus on the occipital arteries.

**Methods:**

90 treatment-naïve patients with clinically and/or histologically proven GCA and/or PMR (51 GCA, 20 PMR, 10 GCA-PMR) were included in the study. All patients underwent contrast-enhanced, fat-suppressed, high-resolution black blood 2D T1-weighted spin echo imaging at 3T MRI. Images were read by three different readers independently. Temporal and occipital arteries were assessed regarding vasculitic affection. Circumferential mural hyperenhancement and thickening of the vessel wall ≥ 600 μm was considered positive for vasculitis.

**Results:**

9/90 (10%) of all patients revealed inflammatory changes of the occipital artery only. Prevalence of isolated inflammatory affection of occipital artery was even higher in the GCA subgroup with 7/51 (14%) patients.

**Conclusion:**

14% of GCA patients and 10% of GCA-PMR patients present with signs of inflammation of the occipital artery only. Since the occipital artery is not accessible to routine ultrasound examination, MRI renders incremental value in the diagnosis of GCA and PMR patients.

## Introduction

Giant Cell Arteritis (GCA) and Polymyalgia Rheumatica (PMR) are systemic inflammatory disorders predominantly affecting elderly individuals over the age of 50, characterized by granulomatous inflammation of medium and large arteries [1]. Some authors suggest that GCA and PMR may not be distinct conditions but exist on a continuum [2]. Patients may also present with manifestations of both conditions simultaneously (GCA-PMR) [3]. The hallmark of GCA is the inflammatory involvement of branches of the external carotid arteries, especially the temporal arteries, leading to cranial symptoms such as headache, jaw claudication, and visual disturbances [4]. However, diagnosis can be challenging, particularly in cases with non-specific symptoms or inconclusive temporal artery biopsy results [3].

While the temporal artery has been the primary focus in diagnosing GCA, the occipital artery, despite its frequent involvement, has been relatively underexplored. This may be due to its less accessible location and the historical reliance on clinical and biopsy findings, which tend to prioritize temporal artery inflammation [5]. The advent of advanced imaging techniques, such as magnetic resonance imaging (MRI), has provided new insights into the inflammatory involvement of extracranial arteries, including the occipital artery [6]. MRI offers high-resolution visualization of arterial walls without radiation exposure, making it particularly useful in detecting inflammation in arteries that are less accessible to other diagnostic methods. While [¹⁸F]Fluorodeoxyglucose positron emission tomography (FDG-PET) can detect systemic inflammation, its cost, radiation exposure, and limited availability favor MRI as a more practical alternative in many cases. Furthermore, FDG-PET can only be used to detect inflammatory changes in the scalp arteries if the scanner provides image quality with sufficient spatial resolution to reliably assess these delicate vessels.

Current guidelines recommend early imaging to support the clinical diagnosis of GCA. Ultrasound, given its availability and cost-effectiveness, is recommended as the first-line diagnostic modality, with MRI or FDG-PET as alternatives [7].

In this short communication, we analyze the pattern of cranial artery involvement and the prevalence of isolated occipital artery inflammation on MRI in patients diagnosed with GCA, PMR and GCA-PMR.

## Methods

### Patient population

Approval for the study from the Ethics Committee of the University Hospital Wuerzburg has been received under the designation 109/19-am in 2019. The study was conducted in adherence to the principles of the Declaration of Helsinki. Following IRB approval and after written informed consent, 90 treatment-naïve patients (56 female, 34 male, mean age 71 years, SD 9) with clinically and/or histologically diagnosed GCA or clinically diagnosed PMR or clinically and/or histologically diagnosed GCA-PMR and MRI of the scalp arteries between July 2018 and January 2023 were included in the trial. Exclusion criteria included an age of < 50 years, uncertain diagnostic criteria, and an existing or ongoing therapy with immunosuppressants. The patients were retrospectively identified from a prospectively collected dataset from the University Hospital Wuerzburg. Clinical diagnosis was established by a rheumatologist or ophthalmologist based on clinical criteria and the 2022 American College of Rheumatology/EULAR Classification Criteria for Giant Cell Arteritis [8] respectively the 2012 provisional classification criteria for polymyalgia rheumatica [9]. All patients were treatment-naïve at the time of MRI.

### Imaging protocol

MRI was performed on a 3-Tesla scanner (MAGNETOM Prisma, Siemens Healthineers), using a dedicated 64-channel head coil. Gadolinium contrast-enhanced, fat-suppressed T1-weighted spin echo sequences were acquired in a transversal plane with a submillimeter spatial resolution of 195 × 260 μm (repetition time (TR)/echo time (TE) 500/19 ms, field of view 220 × 220 mm^2^, acquisition matrix size 1024 × 768 voxels, pixel-bandwith = 96 Hz/px). Eleven slices with a slice thickness of 3 mm and slice distance of 3 mm covered an examination volume stretching over 63 mm. The sequence was initiated ∼ 1 min after intravenous injection of a gadolinium-containing contrast agent (Dotagraf^®^, 0,5 mmol/ml).

### Image analysis

Images were analyzed by three readers independently. One reader was an expert reader (ER) with 7 years of experience in reading vessel wall imaging studies. Two readers were inexperienced readers (IR1 and IR2), medical students who underwent specific training in interpreting vessel wall imaging including side-by-side analysis of 50 vessel wall MRI studies together with a board-approved radiologist and a board-approved neuroradiologist.

All three readers individually evaluated the superficial temporal arteries (main trunk, frontal and parietal branch) and the occipital arteries, both on the right and the left side, regarding inflammatory affection. According to the literature, circumferential contrast enhancement and thickening of the vessel wall > 600 μm was rated positive for inflammation [10]. The temporal artery was considered positive for inflammation if at least one segment met the criteria for a positive finding. Discordant results were reviewed and discussed together and consensus reached by agreement between the raters.

### Statistical analysis

Prism (version 8.4.2 (GraphPad, San Diego, CA, USA)) was used for statistical analysis. Descriptive statistics were used. Continuous variables are presented as means and standard deviations and categorical variables as percentages. Fleiss’ kappa was calculated for comparison of agreement between the raters.

## Results

**Fig. 1:**
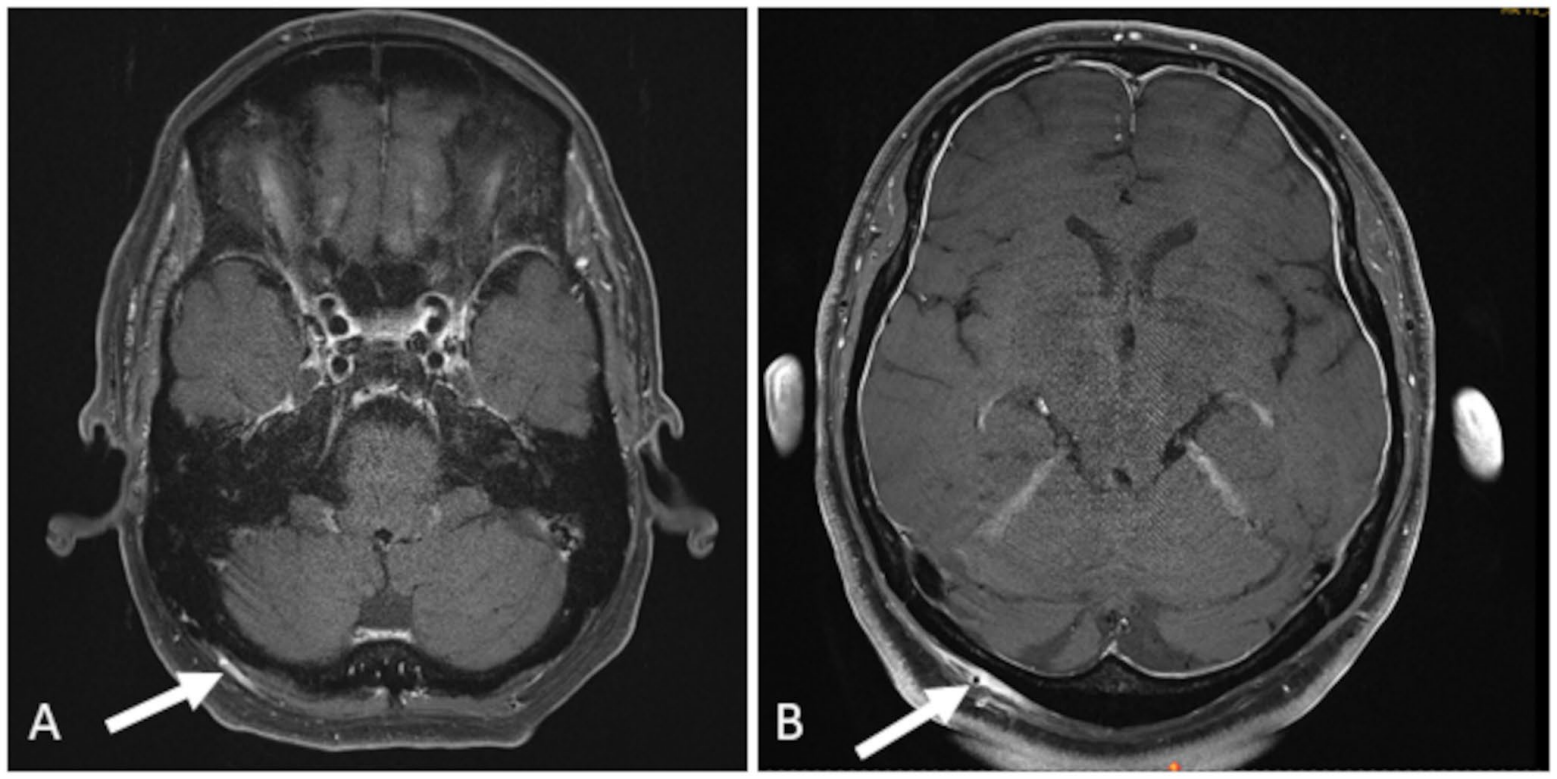
Isolated inflammatory affection of the occipital artery High resolution MRI shows vivid enhancement both of the vessel wall of the right occipital artery and of the surrounding perivascular soft tissue (**A**, **B**, arrow) in two GCA patients. The superficial temporal arteries are unremarkable in both patients

A total of 90 patients with clinically diagnosed GCA, PMR and GCA-PMR were included in the trial. 51/90 (57%) patients were diagnosed with GCA, 20/90 (22%) were diagnosed with PMR, 10/90 (21%) patients presented with clinical characteristics of both GCA and PMR (GCA-PMR). 53/90 (59%) of all patients with diagnosis of GCA, PMR or GCA-PMR showed inflammatory changes of any of the scalp arteries on contrast-enhanced high-resolution MRI (left superficial temporal artery only: 1/90 (1%); right occipital artery only: 4/90 (4%); left occipital artery only: 3/90 (3%); bilateral occipital artery: 2/90 (2%); superficial temporal artery and occipital artery, each of at least one side: 43/90 (48%)). 41/51 (80%) of patients with GCA showed signs of inflammation of any of the scalp arteries (left superficial temporal artery only: 1/51 (2%); right occipital artery only: 3/51 (6%); left occipital artery only: 3/51 (6%); bilateral occipital artery: 1/51 (2%); superficial temporal artery and occipital artery, each of at least one side: 33/51 (65%)). 2/20 (10%) of patients with PMR showed signs of inflammation of the superficial temporal artery and the occipital artery, each of at least one side. 10/19 (53%) of patients with GCA-PMR showed signs of inflammation of any of the scalp arteries (right occipital artery only: 1/19 (5%); bilateral occipital artery: 1/19 (5%); superficial temporal artery and occipital artery, each of at least one side: 8/19 (42%)). Table 1 summarizes the pattern of involvement of the scalp arteries for each diagnosis (GCA, PMR, GCA-PMR). Figure 1 shows contrast-enhanced MRI of two GCA patients with isolated inflammatory involvement of the occipital artery.

**Table 1:** Pattern of involvement of the scalp arteries in patients with GCA and/or PMR

Diagnosis (total *n* = 90)	Signs of inflammation on contrast enhanced high resolution MRI						
	superficial temporal artery only			occipital artery only			superficial temporal artery and occipital artery, each of at least one side
	right	left	bilateral	right	left	bilateral	
GCA (*n* = 51)	0	1	0	3	3	1	33
PMR (*n* = 20)	0	0	0	0	0	0	2
GCA-PMR (*n* = 19)	0	0	0	1	0	1	8

The table shows the pattern of involvement of the scalp arteries for each diagnosis (GCA, PMR, GCA-PMR) on contrast-enhanced high-resolution MRI

Overall, concordance rates were excellent for all analysed vessel segments, with no significant differences observed between ERs and IRs. For the comparison of results for the bilateral occipital arteries, inter-rater reliability among the three readers yielded significant concordance (*p* < 0.001, kappa 1.00, 95% confidence interval 1.00–1.00).

## Discussion

Our study highlights the significant role of MRI in diagnosing GCA and silent GCA in PMR phenotype, particularly in detecting isolated inflammatory involvement of the occipital artery - a phenomenon that has been relatively underexplored in the literature thus far. Previous data have already shown that there is a significant number of patients with occipital artery involvement [11] and atypical manifestations of GCA, characterized by sparing of the temporal arteries and instead inflammatory involvement of other arterial structures of the head, such as the facial artery and the occipital artery [12]. One study demonstrated that in a GCA cohort, 31.2% of patients showed inflammatory involvement of the occipital artery, and that 18.2% of GCA patients with a negative ultrasound of the temporal artery exhibited inflammatory changes in the ultrasound of the facial artery or the occipital artery [12]. However, there is little data in the existing literature regarding the specific involvement patterns of the scalp arteries and regarding the proportion of patients with isolated inflammatory involvement of the occipital artery. Clinical symptoms usually depend on the location of the inflamed vessels. Similar to typical temporal arteritis, patients with inflammatory involvement of the facial artery typically report jaw claudication, while those with inflammatory involvement of the occipital artery generally complain of occipital headaches and nuchal pain, along with swelling and tenderness of the occipital artery [11–13]. Although it is technically possible to biopsy the occipital artery [14, 15], this is not part of the standard workup. Instead, the biopsy is usually performed on the temporal artery due to its easier accessibility and the more familiar surgical technique [14]. Patients with isolated affection of the occipital artery would be missed by standard temporal artery biopsy but can be identified through ultrasound examination [12]. However, the anatomical course of the occipital artery along the back of the head, particularly considering the usual presence of hair in this area, makes it difficult to access for ultrasound examination. Although studies have shown that the occipital artery is at least partially accessible by ultrasound [11, 12], ultrasound examination of the occipital artery is not routinely included in standard ultrasound protocols for the assessment of large-vessel vasculitis in many centers, including our Vasculitis Center at the University Hospital of Wuerzburg.

Integrating MRI into the diagnostic workup for GCA and PMR presents both opportunities and challenges. While MRI has demonstrated considerable promise in identifying inflammatory changes of the cranial arteries with comparable diagnostic accuracy to ultrasound [16], its accessibility and cost may limit widespread adoption compared to ultrasound as a more readily available and cost-effective modality. Furthermore, it has been shown that ultrasound detects vascular changes more frequently than MRI when not only the scalp arteries but all supra-aortic arteries are taken into account, supporting its role as a crucial part of a comprehensive diagnostic workup [17].

Our findings suggest that MRI should be considered an additional tool in the diagnostic workup for suspected GCA or silent GCA within the PMR phenotype, particularly in or reserved for cases with inconclusive temporal artery biopsy results or discrepancies between biopsy, ultrasound findings, and clinical symptoms. This targeted application not only optimizes the use of MRI but also enhances diagnostic accuracy in complex cases. To better integrate MRI into routine practice, it is essential to establish clinical protocols that clearly define when MRI should be employed, especially in scenarios where ultrasound may be less effective. Furthermore, increasing clinician awareness of the benefits of MRI and exploring cost-effective solutions - such as a streamlined, focused examination protocol - can improve accessibility, ultimately leading to more accurate and timely diagnosis of GCA.

Our observation of isolated occipital artery inflammation in a notable proportion of patients challenges the prevailing view that GCA primarily affects the temporal arteries. Although temporal artery involvement remains a hallmark of GCA, our findings suggest a significant number of patients exhibit atypical manifestations that spare the temporal arteries, potentially missed by standard diagnostic approaches. The utility of MRI in detecting isolated occipital artery inflammation carries significant clinical implications. Given the limitations of temporal artery biopsy, such as sampling error and false-negative results [18], MRI emerges as a valuable complementary tool for identifying extracranial arterial involvement in GCA and PMR. By facilitating the prompt identification of patients with active inflammation, MRI can help initiate appropriate therapy and reduce the likelihood of missed diagnoses. This aligns with recent literature advocating for the incorporation of an independent reference diagnosis into the diagnostic algorithm for GCA, particularly in cases where traditional modalities are inconclusive [18]. Notably, FDG-PET serves as a limited alternative to MRI due to its restricted resolution and availability, relatively high costs, and associated radiation exposure.

Additionally, our findings underscore the importance of considering the heterogeneous presentations of patients with GCA and/or PMR and the necessity of a multidisciplinary approach to diagnosis and management [1]. Clinicians should maintain a high index of suspicion for GCA and/or PMR in patients presenting with non-specific clinical symptoms, even in the absence of temporal artery abnormalities [19].

Despite the strengths of our study, including a relatively large sample size and systematic MRI evaluation, several limitations warrant consideration. The retrospective design inherently carries the risk of selection bias and incomplete data capture. Additionally, the lack of a control group limits our ability to assess the specificity of isolated occipital artery inflammation as a diagnostic marker for GCA and PMR. Future prospective studies that incorporate longitudinal follow-up and comparisons with healthy controls are warranted to further elucidate the clinical significance of isolated occipital artery inflammation in GCA and PMR. Another limitation is that we did not conduct targeted ultrasound of the occipital artery in the presented cohort, which means we cannot determine how many patients we might have identified as positive for vasculitis without the use of MRI, but with a more comprehensive ultrasound examination. Moreover, while we focused on the isolated inflammatory involvement of the occipital artery in this study, it remains to be determined whether other vessels within the GCA spectrum could also be affected by isolated inflammatory changes, potentially resulting in patients being overlooked by conventional diagnostic algorithms.

In conclusion, our study provides novel insights into the role of MRI in diagnosing GCA and PMR, particularly in detecting isolated inflammatory involvement of the occipital artery. By highlighting the prevalence, variability and clinical significance of extracranial arterial involvement patterns, our findings underscore the importance of a comprehensive diagnostic approach that integrates clinical and laboratory assessments, and, when inconclusive, various imaging modalities. Moving forward, further research is needed to validate the diagnostic utility of MRI and optimize the diagnostic algorithm for GCA and PMR.

## Data Availability

In line with the principles of transparency and reproducibility, we are committed to sharing the data that support the findings of this study. The datasets generated and analyzed during the current study are available from the corresponding author upon reasonable request. Data will be provided in accordance with ethical guidelines and legal requirements, ensuring participant confidentiality and privacy are protected. Where applicable, anonymized data may be shared in publicly accessible repositories following acceptance of the manuscript.
